# An Improved Score for the Evaluation of Mucosal Healing in Inflammatory Bowel Disease—A Pilot Study

**DOI:** 10.3390/jcm12041663

**Published:** 2023-02-19

**Authors:** Lidia Neamți, Tudor Drugan, Cristina Drugan, Ciprian Silaghi, Lidia Ciobanu, Alexandra Crăciun

**Affiliations:** 1 Department of Medical Biochemistry, “Iuliu Hațieganu” University of Medicine and Pharmacy, 400012 Cluj-Napoca, Romania; 2 Gastroenterology Department, Regional Institute of Gastroenterology and Hepatology “O. Fodor”, 400000 Cluj-Napoca, Romania; 3 Department of Medical Informatics and Biostatistics, “Iuliu Hațieganu” University of Medicine and Pharmacy, 400012 Cluj-Napoca, Romania

**Keywords:** inflammatory bowel disease, Crohn’s disease, ulcerative colitis, fecal calprotectin, endoscopic activity

## Abstract

Inflammatory bowel diseases are chronic conditions characterized by periods of remission, alternating with episodes of exacerbation, in which the primary therapeutic target is mucosal healing. Although colonoscopy is currently considered the gold standard for assessing disease activity, it presents a significant number of disadvantages. Over time, various inflammatory biomarkers have been proposed to detect disease activation, but current biomarkers have many limitations. Our study aimed to analyze the most commonly used biomarkers for patient monitoring and follow-up both independently and taken together as a group, in order to propose an improved activity score that more accurately reflects the changes occurring at the intestinal level, in order to limit the number of colonoscopic interventions. By applying logistic regression as a method of statistical analysis to the retrospectively collected data, we obtained an easy-to-calculate improved score that quantifies the chance that a given patient may be in remission or in a period of endoscopic activity. To achieve a widely accessible score that is easily accessible in clinical practice, we have included only the most commonly used clinical and biological parameters.

## 1. Introduction

Inflammatory bowel diseases (IBD) are chronic conditions that affect the gastrointestinal tract and are characterized by periods of remission, alternating with episodes of exacerbation [1]. These disorders include Crohn’s disease (CD) and ulcerative colitis (UC). Although the mechanisms that trigger the disease onset are not yet sufficiently elucidated, their pathogenesis is similar. These conditions generally start at a young age, causing disabilities in the field of work and being responsible for a significant increase in the economic and social burden [2].

Given the recurrent evolution of exacerbation episodes, these patients require a continuous follow-up, with periodic evaluation to immediately detect and treat any possible recurrence. The therapeutic target is mucosal healing, which is a good predictor of disease remission [3]. Currently, colonoscopy is used to objectivate the presence and extent of mucosal lesions. This investigation is considered the gold standard for assessing disease activity. However, colonoscopy is an invasive, expensive, time-consuming procedure, that involves a significant risk of complications, especially during the active period of the disease. Therefore, monitoring disease evolution through repeated colonoscopies is not considered appropriate [4].

Various clinical activity scores have been developed for the long-term follow-up of disease progression [5,6,7]. However, some of them showed a poor correlation with endoscopic activity [8]. Over time, since mucosal lesions are often significant, even in the absence of clinical manifestations, different inflammatory biomarkers have been proposed, in order to detect disease reactivation as quickly and accurately as possible. Currently, the most widely used biomarkers are fecal calprotectin (FC) and C-reactive protein (CRP).

Fecal calprotectin is a protein secreted by neutrophils that, to date, best correlates with intraluminal changes. However, its major drawbacks are the significant intra-individual variability [9], as well as its dependence on the affected bowel segment [10]. Taken together, these inconveniences lead to a decrease in the diagnostic accuracy of this biomarker.

C-reactive protein is an acute-phase protein that is highly sensitive to inflammation. However, in the case of IBD patients with episodes of exacerbated disease activity, circulating CRP levels may often increase insufficiently for an accurate evaluation of mucosal lesions [11].

As a result of the limitations of current biomarkers, finding non-invasive ways to evaluate intestinal changes before the appearance of clinical symptoms would be of real benefit to both patients and physicians. If effective, this strategy might also reduce the economic and social burden imposed by disease reactivation.

Our study aimed to analyze different markers of inflammation used during the process of disease monitoring and patient follow-up in inflammatory bowel diseases. Our approach was to evaluate them by logistic regression, both independently and as a group, in order to propose an activity score that more accurately reflects the changes occurring at the intestinal level.

## 2. Materials and Methods

An analytical retrospective study was designed and approved by the ethics committee of the University of Medicine and Pharmacy “Iuliu Hațieganu” of Cluj-Napoca.

All included subjects had a confirmed diagnosis of IBD, based on clinical, colonoscopic, histopathological and imaging criteria. At the time of presentation, they had performed a colonoscopy and a fecal calprotectin measurement had been scheduled at maximum 3 days prior to the endoscopic examination. Other laboratory assays had been performed the week before the colonoscopy, preferably close to the endoscopic investigation. At the moment of inclusion, the patients were on their chronic treatment.

Excluded subjects were those with indeterminate colitis, a history of bowel surgery for other causes than IBD, pregnant women, or patients with other conditions susceptible of influencing the levels of inflammatory markers (such as infections occurring less than a month before presentation, a history of recent trauma or surgery in the previous month, evolving neoplastic or autoimmune disease).

Medical files were retrospectively collected from the Regional Institute of Gastroenterology and Hepatology Cluj-Napoca hospital database. The search was carried out over a period of 6 years, between January 2015 and December 2020.

The classification of patients according to the state of clinical and endoscopic activity was based on previously validated severity scores, widely used in clinical practice [7].

The clinical characteristics of the studied patients were evaluated using Montreal score [12].

For patients with CD, clinical activity was evaluated by the “Crohn’s disease activity index” (CDAI) [7]. Patients with a score of < 150 points were considered in remission, those situated between 150–220 points had mild activity, those between 220–450 points had moderate activity and a score of > 450 points was considered indicative of severe activity.

For patients with UC, the partial Mayo score [7] was used as follows: a score of <2 points was considered indicative of disease remission, a score between 2–4 points was assigned to mild activity, between 5–7 points to moderate activity, and a score > 7 points was an indicator of severe activity.

Mucosal healing was defined in the case of a SES-CD score < 2, and a Mayo score of 0, respectively.

The “Simple endoscopic score for Crohn’s disease” (SES-CD) [7] was used to evaluate the endoscopic activity of patients with CD. The degrees of activity were divided as follows: <2 points—remission, between 3–6 points—mild activity, between 7–15 points—moderate activity, and >16 points—severe activity. The endoscopic Mayo score [7] was used to evaluate endoscopic changes in UC patients. A score of 0 points was considered suggestive of disease remission, 1 point indicated mild activity, 2 points—moderate activity, and 3 points suggested severe disease activity.

The inflammation markers frequently used to evaluate these patients were also registered. Thus, for each patient, the values of the following parameters were recorded: the number of leukocytes (reference range 4000–10,000/μL), erythrocyte sedimentation rate (ESR) at 1 and 2 h (reference ranges < 11 mm and < 14 mm, respectively), C-reactive protein (CRP, reference range 0–1 mg/dL) and fecal calprotectin (reference range < 50 μg/g).

For statistical analysis, we used SPSS (IBM Corp. Released 2017, Version 25.0. Armonk, NY, USA).

To assess the normality of the distribution of quantitative variables, we used the Kolmogorov–Smirnov test. The mean and standard deviation were calculated for normally distributed variables and the median was used for variables not following a normal distribution.

The differences between groups were evaluated using non-parametric tests or the Student test, depending on data distribution. ROC (receiver operating characteristic) curves were performed to measure the ability of each parameter to predict changes at the intestinal level and the corresponding areas under the curve (AUC) was measured. Logistic regression was used to combine the effect of different parameters, currently used to monitor inflammatory bowel diseases.

For each parameter, a value of “0” or “1” was assigned, depending on whether it was situated below, or above the cut-off values, respectively. Following regression analysis, relative weights were established for each parameter and then included in the calculation of the proposed diagnostic score.

A *p*-value < 0.05 was considered significant.

## 3. Results

A total of 209 subjects were included in this study. Among them, 72 patients were diagnosed with CD and 137 with UC. In 23 subjects, the values of ESR were not available, and in one patient, the value of CRP was not recorded. Otherwise, the other variables were collected according to the study protocol.

The clinical characteristics of the patients are summarized in Table 1.

Non-invasive parameters for the evaluation of IBD activity, according to the endoscopic classification as activity or remission episodes, are presented in Table 2.

The median FC values were 410 μg/g for CD and 740 μg/g for UC, whereas the median CRP values were 0.85 mg/dL in CD and 0.50 mg/dL in UC. Statistically significant differences (*p* < 0.05) were found in both FC and CRP values, between patients in remission and those in the active disease phase, for both IBD types.

Furthermore, we classified the patients in activity periods, according to the degree of endoscopic severity, as mild, moderate and severe subgroups. Statistical tests were performed to highlight eventual differences between adjacent categories. These results are summarized in Table 3 and Table 4 for Crohn’s disease and ulcerative colitis, respectively.

Following, the monitored parameters were analyzed according to the diagnostic performance. Following the evaluation of the ROC curves by the AUC, regardless of the condition, a value of 0.903 [0.853–0.952] was observed for FC when analyzing the patients with disease remission and, taken together, those with different activity subgroups. The ability of FC to differentiate between adjacent categories decreased with the increasing severity of the acute episode. Therefore, the AUC was 0.833 [0.745–0.921] between patients in remission and those with mild activity, 0.664 [0.566–0.761] between those with mild and with moderate activity and 0.528 [0.427–0.629] between those with moderate and with severe activity.

The same pattern of variation was observed for the other analyzed parameters. AUC values between patients in remission and those with disease activity were 0.763 [0.692–0.835] for CRP, 0.828 [0.725–0.921] for CDAI, 0.874 [0.800–0.928] for the Mayo score, 0.743 [0.641–8.45] for ESR at 1 h and 0.529 [0.405–0.653] for leukocytes.

For FC, the cut-off value was set at 205 μg/g and for CRP, at 1 mg/dL. These values were chosen by the Youden index method, in order to obtain the best sensitivity and specificity.

Table 5 shows the sensitivity, specificity, the positive and negative predictive values, along with the 95% confidence interval for the monitored parameters.

We then used logistic regression to establish the weight of each parameter within an activity score, that combines clinical severity scores and biological markers. Such a score would increase the diagnostic performance of individual parameters and would also improve the classification power established solely by logistic regression. Table 6 presents the regression coefficients obtained by logistic regression and those proposed for further calculation of the activity score, obtained by the transformation of the former into absolute values.

We obtained the following calculation formulas for the proposed activity scores, corresponding to Crohn’s disease (Equation (1)) and ulcerative colitis (Equation (2)), using only the linear component of the logistic regression, in order to simplify their use by the practicing physicians:

(1) $$\mathrm{CD}\;\mathrm{Score}=\frac{\mathrm{CDAI}}{1000}+\frac{\mathrm{FC}}{500}+\mathrm{CRP}$$

(2) $$\mathrm{UC}\;\mathrm{Score}=\frac{\mathrm{Mayo}\;\mathrm{score}}{4}+\frac{\mathrm{FC}}{100}+\frac{\mathrm{CRP}}{4}$$

where CD = Crohn’s disease, CDAI = Crohn’s disease activity index, CRP = C-reactive protein (mg/dL), FC = fecal calprotectin (μg/g) and UC = ulcerative colitis.

**Figure 1:**
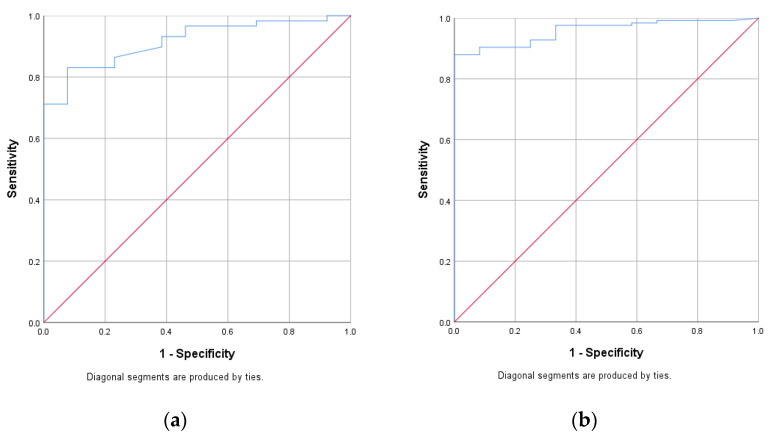
ROC (receiver operating characteristic) curve corresponding to the proposed activity score in (**a**) Crohn’s disease and (**b**) ulcerative colitis.

The ROC curves corresponding to the activity scores developed for CD and UC are presented in Figure 1. For CD, the validation of the score on the studied patients produced an AUC of 0.917 [0.853–0.983], whereas, for UC, the AUC was 0.958 [0.924–0.992]. Using the Youden coefficient, we set the threshold level at 1 for the CD score and at 2 for the UC score. For CD, this corresponds to a sensitivity of 0.831 and a specificity of 0.923 (*p* < 0.001), while for UC, it implies a sensitivity of 0.904 and a specificity of 0.917 (*p* < 0.001).

**Figure 2:**
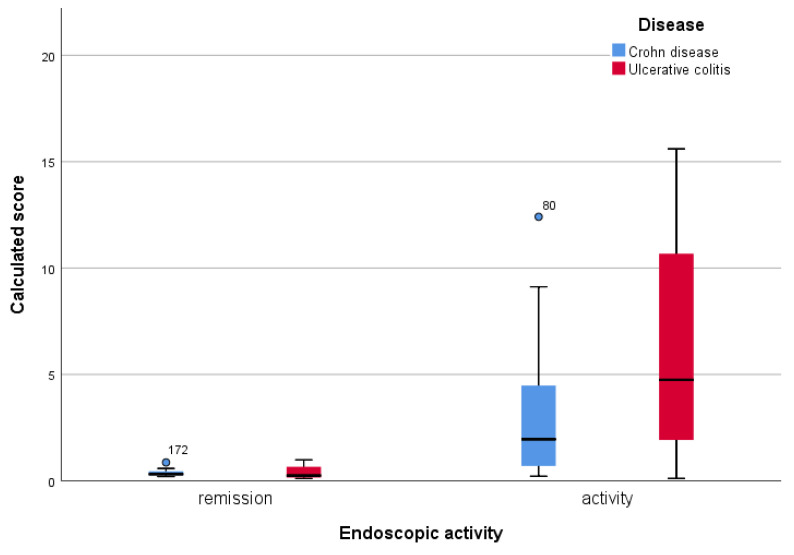
Median values of the activity score calculated according to the degree of endoscopic activity, for each of the studied conditions (◦ mild outlier).

Using the Kolmogorov–Smirnov test, we noticed that the obtained scores did not have a normal distribution (*p* < 0.001). As a result, we compared the distribution and median values for each condition, in remission versus activity. Table 7 shows the median and the 25% and 75% percentiles, while Figure 2 shows the median of the calculated score for each disease.

**Figure 3:**
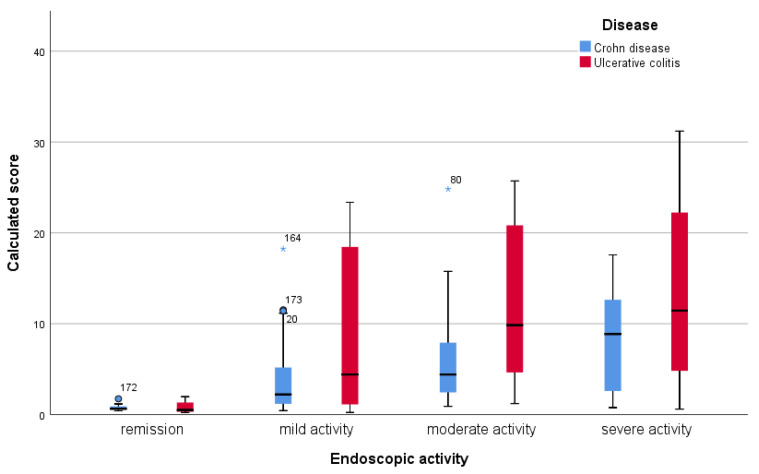
Median values of the activity score calculated for each subcategory of endoscopic activity, in Crohn’s disease and ulcerative colitis. (◦ mild outlier, * extreme outlier).

Taking into account the subcategories of endoscopic activity, we obtained the values indicated in Table 8 which are graphically represented in Figure 3.

## 4. Discussion

In this study, we evaluated the diagnostic performance of several inflammatory parameters currently used for patient follow-up in inflammatory bowel diseases. Considering the specific limitations and inconveniences of each individual parameter, resulting in poor diagnostic capability, we used logistic regression to develop an activity score that would allow a significant increase in diagnostic sensitivity and specificity. To avoid the impact of treatment on the symptoms and biological parameters, all included patients were already on their chronic treatment before any adjustments had been made to it.

Previous studies have shown significant differences between the values of clinical scores or biological markers and the observed endoscopic intestinal changes [13,14]. For example, our study comprised several patients with a clinical score suggestive of remission, but with endoscopic lesions indicating disease reactivation. This difference between current clinical scores or individual biomarkers and endoscopic findings highlights the need for a non-invasive parameter that more precisely reflects intestinal changes.

Both FC and CRP are biomarkers that allow the differentiation between the remission and activity phases of IBD. However, according to our results, the ability of FC to differentiate between adjacent activity subcategories was found to decrease with the increasing severity of the acute flare.

In line with previously reported data [15], our study reinforces the utility of FC in differentiating between episodes of remission and mild activity. However, in our patients, this biomarker failed to indicate significant differences between moderate and severe activity, unlike in the study conducted by Chen et al. [15]. Compared to the mentioned study, our results did show significant differences between the values of FC in patients with mild versus moderate activity, without reaching statistical significance between the other subcategories, neither for CD nor UC.

As for CRP, according to our results, this biomarker could differentiate between patients in remission and those with mild activity, in both studied conditions, though its circulating levels showed no significant differences between the other subcategories.

Regarding the clinical activity scores, we noticed that the CDAI score was significantly lower in CD patients with remission, compared to those with mild activity. However, the Mayo score could not differentiate between these two categories, being significantly lower only in CU patients with mild activity, compared to those with moderate activity.

In the present study, the highest specificity was obtained for CRP and FC. However, these results must be interpreted cautiously, because at the chosen cut-off value, there were no patients in endoscopic remission with a CRP or FC level above the cut-off value. Consequently, in our study, the highest sensitivity was obtained for ESR at two hours, while the highest specificity was obtained for the CDAI score in patients with CD, and the leukocyte count of in those with UC.

Consistent with other studies, in our patients, the CDAI score achieved the best specificity [14,15]. Regarding FC, previous studies have obtained different diagnostic performances, with a sensitivity between 50–96% for CD and 11–100% for UC and a specificity between 52–100% for CD and 50–100% for UC [16].

In our study, the leukocyte count was excluded from the proposed activity score because no significant differences were found between its values during remission and activity phases. We also excluded ESR values from the calculation of the diagnostic score, which led to an increase in the AUC.

Some previously published studies tried to combine different parameters, to improve the assessment of intestinal changes. Thus, Langhorst et al. [17] tried to differentiate patients in activity from those in remission by designing an activity score that combined the CDAI score, the level of circulating CRP and the values of three fecal parameters: calprotectin, lactoferrin and polymorphonuclear neutrophil elastase. The score was considered positive if it met at least two of the following conditions: 1) the CDAI score was increased, 2) the level of CRP was above the cut-off value (7 mg/L) and 3) at least two fecal parameters were raised. This score’s sensitivity (85.2%) and specificity (88%) were superior to those of other parameters when both CD and UC patients were considered. However, in the case of CD, the diagnostic performance of the new score remained inferior to that of FC. Moreover, the measurement of three fecal markers, alongside CRP and a clinical score, proved to be a challenging and costly procedure for patient follow-up.

In another study, several clinical and biological parameters (the Harvey–Bradshaw index—HBI, CDAI, CRP, fecal calprotectin) were evaluated as potential indicators of endoscopic remission in CD patients, treated with anti-tumor necrosis factor (TNF). The best diagnostic performance was obtained using the following score: fecal calprotectin (μg/g) + 60 × HBI, which had a sensitivity of 86% and a specificity of 82%. However, its diagnostic accuracy was not significantly different from that of fecal calprotectin alone, which had a sensitivity of 84% and a specificity of 74% [14]. Moreover, the paper did not mention explicitly how this score was obtained, thus limiting the application of this approach.

A recent study by de Bruyn et al. used binary logistic regression and combined several biomarkers of inflammation, such as serum neutrophil count, CRP, cathelicidin LL-37 and chitinase 3-like 1 to increase the diagnostic performance in the evaluation of mucosal healing in UC patients, undergoing anti-TNF treatment. Their proposed response index had a sensitivity of 54% and a specificity of 92% [18]. The major drawback of this approach was the inclusion of infrequent biomarkers, unlike those chosen in our study, which are part of the routine diagnostic protocols and can be assayed by any hospital laboratory.

Chen et al. used multiple linear regression to combine several clinical and biological parameters (FC, CDAI, clinical activity index—CAI, CRP, ESR, procalcitonin) in order to obtain an activity score that best predicts endoscopic activity in patients with UC or with the ileocolonic form of CD. Performing the following calculations: 0.2 × FC + 50 × CAI and 0.8 × FC + 4.6 × CDAI, for UC and CD, respectively, resulted in a sensitivity and specificity above 90% for both disease categories [15].

In order to detect inflammation at the intestinal level, other studies tried to combine biological markers, such as FC, with a questionnaire for the evaluation of the quality of life (i.e., the “Short Inflammatory Bowel Disease Questionnaire”) [19], or the FC values with either an activity index or the CRP levels [20]. In both cases, higher diagnostic performance was achieved, compared to the individual analysis of each parameter.

Although the score developed in the present study showed higher diagnostic performance than each individual parameter, its validation is mandatory, before it could be used in the clinical setting. Also, cost-effectiveness studies are needed to estimate the percentage of endoscopic investigations that could be avoided, using this activity score. One such study, performed for the evaluation of FC alone, concluded that its assay could avoid an important number of endoscopic interventions and reduce the cost by 1010 USD/patient, over a period of 18 months [21].

A major limitation of our study lies in its retrospective character. It is possible that the relatively low incidence of both diseases in our study may be due to an insufficient awareness about those disorders among primary care physicians and, subsequently, an underdiagnosis of inflammatory bowel disorders in our region. Furthermore, for a more precise analysis of the monitored parameters, the subjects were divided into four groups, according to the degree of endoscopic activity (remission, mild, moderate and severe activity), resulting in a relatively small number of patients in each subgroup. We may speculate that better diagnostic performance of the proposed activity scores might be observed with an increase in the number of studied patients, for both conditions. Also, in patients with ileal Crohn’s disease, colonoscopy cannot assess the mucosal changes in the small intestine, which may lead to misclassification of the subjects in remission or activity phases. Finally, we must consider the degree of subjectivity of current clinical and endoscopic scores, besides the performance of endoscopic investigations by different practitioners, which may further interfere with patient classification among the different phases of disease activity.

## 5. Conclusions

In conclusion, although several inflammatory biomarkers may be used for patient follow-up with the purpose of limiting the number of colonoscopies, these parameters cannot entirely replace endoscopic investigations. By applying logistic regression as a statistical analysis method to retrospectively collected data, we obtained a relatively easy-to-calculate score that quantifies the chance that a given patient may be in remission or in a phase of endoscopic activity, for each of the studied diseases. Contrary to other similar activity scores, we only included the most frequently used clinical and biological parameters, in order to obtain an improved score, widely accessible in clinical practice.

## Figures and Tables

**Table 1:** Clinical characteristics of the studied patients.

	Crohn’s Disease	Ulcerative Colitis
Number of patients (*n*)	72	137
Number (%) of male patients	34 (47.2%)	69 (50.4%)
Age at study inclusion (years) (mean ± standard deviation)	38.4 ± 12.7	43.9 ± 17
Age at diagnosis (years) (mean ± standard deviation)	32.8 ± 11.8	37.9 ± 16.3
**Montreal classification**		
**Age at diagnosis (years)**		
● A1 (< 16)	3 (4.2%)	
● A2 (17–40)	51 (70.8%)	
● A3 (> 40)	18 (25%)	
**Disease location**		
● Ileum (L1)	23 (31.9%)	
● Colon (L2)	15 (20.8%)	
● Ileum + colon (L3)	32 (44.4%)	
● Upper gastrointestinal tract (L4)	2 (2.9%)	
● Rectum (E1)		16 (11.7%)
● Distal colitis (E2)		76 (55.5%)
● Extensive colitis (E3)		45 (32.8%)
**Disease behavior**		
● Non-stricturing/non-penetrating (B1)	41 (56.9%)	
● Stricturing (B2)	23 (31.9%)	
● Penetrating (B3)	8 (11.1%)	
● Perianal disease	16 (22.2%)	
● Remission (S0)		29 (21.2%)
● Mild severity (S1)		50 (36.5%)
● Moderate severity (S2)		39 (28.5%)
● Severe severity (S3)		19 (13.9%)

**Table 2:** Description of non-invasive inflammation assessment parameters according to disease type and activity.

	Crohn’s Disease		Ulcerative Colitis	
	Remission (*n* = 13)	Activity (*n* = 59)	Remission (*n* = 12)	Activity (*n* = 125)
**CDAI** **mean ± SD**	70.46 ^1,^** ± 44.55	179.51 ^1,^** ± 109.73		
**Mayo score** **(median)**			0 ^1,^* (0–3)	5 ^1,^* (0–8)
**FC (μg/g)** **(median)**	80 ^1,^* (5–570)	600 ^1,^* (20–2640)	37.50 ^1^ (15–187)	835 ^1,^* (14–2570)
**CRP (mg/dL)** **(median)**	0.44 ^1^ (0.29–0.75)	1.28 ^1,^* (0.29–21.1)	0.42 ^1,^* (0.27–0.47)	0.55 ^1,^* (0.22–14.99)
**Leukocytes/μL** **(mean ± SD)**	7700 ± 2908.78	8780.33 ± 3113.08	7780 ± 2127.39	8218.76 ± 2788.08
**ESR 1 h** **(median)**	18 (3–54)	38 (2–120)	12 ^1,^* (3–36)	28.50 ^1,^* (2–110)
**ESR 2 h** **(median)**	34 (5–88)	56 (4–144)	20 ^1,^* (5–62)	54 ^1,^* (4–142)

CDAI = Crohn’s disease activity index, CRP = C-reactive protein, ESR = erythrocyte. sedimentation rate, FC = fecal calprotectin, *n* = number of patients SD = standard deviation. Results are expressed as the mean ± standard deviation for normally distributed data and as the median (the minimum value—the maximum value) in the case of non-normally distributed variables. ^1^ = there are significant differences between patients in remission, compared to those in the period of disease activation (*p* < 0.05). * independent samples Mann–Whitney *U* test. ** independent samples *t*-test.

**Table 3:** Description of non-invasive parameters, according to the degree of endoscopic activity, in patients with Crohn’s disease.

	Remission (*n* = 13)		Mild (*n* = 28)		Moderate (*n* = 15)		Severe (*n* = 16)
**CDAI (mean)**	70.46	*p* < 0.05 **	144.86	NS **	196.60	NS **	224.13
**FC (μg/g)** **(median)**	80	*p* = 0.001 *	350	*p* < 0.05 *	890	NS *	1085
**CRP (mg/dL)** **(median)**	0.44	*p* < 0.05 *	0.72	NS *	1.25	NS *	5.46
**Leukocytes/μL (mean)**	7700	NS **	8200.35	NS **	9666.66	NS **	8964.37
**ESR 1 h** **(median)**	18	NS *	27	NS *	28	*p* < 0.05 *	58
**ESR 2 h** **(median)**	34	NS *	47	NS *	52	NS *	87.50

CDAI = Crohn’s disease activity index, CRP = C-reactive protein, ESR = erythrocyte sedimentation rate, FC = fecal calprotectin, NS = statistically insignificant, *p* > 0.05. Results are expressed as the mean for normally distributed data and as the median in the case of non-normally distributed variables. * independent samples Mann–Whitney *U* test. ** independent samples *t*-test.

**Table 4:** Description of non-invasive parameters, according to the degree of endoscopic activity, in patients with ulcerative colitis.

	Remission (*n* = 12)		Mild (*n* = 28)		Moderate (*n* = 49)		Severe (*n* = 47)
**Mayo score** **(median)**	0	NS *	1.50	*p* = 0.000 *	5	NS *	6
**FC (μg/g)** **(median)**	37.50	*p* = 0.001 *	377	NS *	820	NS *	960
**CRP (mg/dL) (median)**	0.42	*p* < 0.05 *	0.47	NS *	0.58	NS *	1.21
**Leukocytes/μL (mean)**	7780	NS **	8200	NS **	8079.40	NS **	8378.21
**ESR 1 h** **(median)**	12	*p* < 0.05 *	23.50	NS *	30	NS *	30
**ESR 2 h** **(median)**	20	*p* < 0.05 *	41	NS *	54	NS *	57

CRP = C-reactive protein, ESR = erythrocyte sedimentation rate, FC = fecal calprotectin, NS = statistically insignificant, *p* > 0.05. Results are expressed as the mean for normally distributed data and as the median in the case of non-normally distributed variables. * independent samples Mann–Whitney *U* test. ** independent samples *t*-test.

**Table 5:** Description of the diagnostic performance for predicting endoscopic activity of the studied parameters, for Crohn’s disease and ulcerative colitis.

		FC	CRP	ESR 1 h	ESR 2 h	Leukocytes
**Crohn’s disease**	**CDAI**					
**Se**	54 (48–55)	78 (71–80)	59 (53–59)	87 (82–92)	94 (91–98)	28 (22–33)
**Sp**	92 (64–99)	84 (56–97)	100 (74–100)	36 (13–62)	15 (2–31)	61 (34–84)
**PPV**	97 (86–99)	95 (88–99)	100 (90–100)	87 (82–92)	82 (80–86)	77 (61–90)
**NPV**	30 (21–33)	45 (30–52)	35 (26–35)	36 (13–62)	40 (7–82)	16 (9–21)
**Ulcerative colitis**	**Mayo score**					
**Se**	81 (78–83)	79 (76–79)	40 (37–40)	82 (80–85)	92 (90–95)	20 (17–21)
**Sp**	75 (44–93)	100 (71–100)	100 (71–100)	41 (17–69)	41 (17–67)	83 (53–97)
**PPV**	97 (93–99)	100 (96–100)	100 (93–100)	92 (89–96)	93 (90–96)	92 (79–98)
**NPV**	28 (16–34)	31 (22–31)	14 (9–14)	20 (8–34)	38 (16–61)	9 (5–10)

CRP = C-reactive protein, ESR = erythrocyte sedimentation rate, FC = fecal calprotectin, NPV = negative predictive value, PPV = positive predictive value, Se = sensitivity, Sp = specificity.

**Table 6:** Linear regression coefficients obtained by logistic regression and those proposed for further calculation.

Crohn’s Disease			Ulcerative Colitis		
	Regression Coefficient	Proposed Coefficient		Regression Coefficient	Proposed Coefficient
**CDAI**	0.001	1/1000	**Mayo score**	0.264	¼
**CRP (mg/dL)**	0.718	1.000	**CRP (mg/dL)**	0.265	¼
**FC (μg/g)**	0.002	1/500	**FC (μg/g)**	0.009	1/100

CDAI = Crohn’s disease activity index, CRP = C-reactive protein, FC = fecal calprotectin.

**Table 7:** Description of the activity score according to the degree of endoscopic activity.

				Calculated score			
				Median	Percentile 25	Percentile 75	Comparison
**Type of disease**	**CD**	**Endoscopic activity**	**remission**	0.63	0.54	0.90	*p* < 0.001 *
			**activity**	3.91	1.39	9.03	
	**UC**	**Endoscopic activity**	**remission**	0.50	0.31	1.33	*p* < 0.001 *
			**activity**	9.49	3.84	21.35	

CD = Crohn’s disease, UC = ulcerative colitis * independent samples median and Mann–Whitney *U* test.

**Table 8:** Description of the activity score according to the subcategories of endoscopic activity.

				Calculated score			
				Median	Percentile 25	Percentile 75	Comparison
**Type of disease**	**CD**	**Endoscopic activity**	**Remission**	0.63	0.54	0.90	*p* < 0.001 *
			**Mild activity**	2.21	1.18	5.19	
			**Moderate activity**	4.41	2.42	8.52	
			**Severe activity**	8.87	2.59	12.64	
	**UC**	**Endoscopic activity**	**Remission**	0.50	0.31	1.33	*p* < 0.001 *
			**Mild activity**	4.40	1.12	18.46	
			**Moderate activity**	9.84	4.63	20.82	
			**Severe activity**	11.44	4.63	22.46	

CD = Crohn’s disease, UC = ulcerative colitis. * Independent samples Kruskal-Wallis test.

## Data Availability

No new data were created or analyzed in this study. Data sharing does not apply to this article.
